# Evaluation of *Cassia occidentalis* for *in vitro* cytotoxicity against human cancer cell lines and antibacterial activity

**DOI:** 10.4103/0253-7613.68428

**Published:** 2010-08

**Authors:** Madhulika Bhagat, Ajit Kumar Saxena

**Affiliations:** School of Biotechnology, University of Jammu, Jammu, India

**Keywords:** Antibacterial activity, *Cassia occidentalis*, cytotoxicity

## Abstract

**Objective:**

To evaluate the *in vitro* cytotoxicity and antibacterial properties of Cassia occidentalis (whole plant) via alcoholic, hydro-alcoholic, and aqueous extracts against eight human cancer cell lines from six different tissues and four bacterial strains.

**Material and Methods:**

*in vitro* cytotoxicity against the human cancer cells, cultured for 48h in presence of different concentrations *C. occidentalis* extracts and percentage of cell viability, was evaluated using the sulforhodamine-B (SRB) assay. The antibacterial activity was performed using the standard protocol against bacterial strains.

**Results:**

It was observed that aqueous extract of *C. occidentalis* (whole plant) had more potential than hydro-alcoholic and alcoholic extracts against HCT-15, SW-620, PC-3, MCF-7, SiHa, and OVCAR-5 human cancer cell lines at 100, 30, and 10 μg/ml in a dose-dependent manner. The hydro-alcoholic extract showed potential against *Bacillus subtillis*.

**Conclusion:**

The plant can be explored for the possible development of lead molecules for drug discovery.

## Introduction

Approximately 80% of the world’s population still relies on traditional medicines for the treatment of common illnesses.[1] It is observed that management of cancer and infectious diseases always require search for new drugs. Although numerous drugs are currently in use for cancer chemotherapy, they exhibit cell toxicity, induces genotoxic, carcinogenic, and teratogenic effects in non-tumor cell.[2] These side effects limit use of chemotherapeutic agents despite their high efficacy in treating target malignant cells. Similarly, antibacterial drug resistance has been a therapeutic problem.[3] Therefore, the search for novel drugs that are both effective and non-toxic bioactive plant products has been increased.[4] *Cassia occidentalis* (Leguminosae) plant has been extensively used in indigenous and folk-lore medicine system. In Indian system of medicine the plant has been documented as thermogenic, puragative, expectorant, diuretic, and used in the treatment of leprosy, erysipelas, ulcers, cough, bronchitis, constipation, flatulence, dyspepsia, menstrual problems, tuberculosis, and anemia.[5] Reports for its immunosuppressive,[6] antimutagenic,[7] anti-inflammatory,[8] anti-dermatophyte,[9] antibacterial,[10] antiplasmodial,[11] anti-fertility,[12] antimalarial,[13] and antidiabetic[14] activities had been published. The leaf extracts of the plant have been reported to repair, protect, and normalize the liver functions.[15] Considering the therapeutic values of *C. occidentalis*, the present work was undertaken to evaluate the **in vitro** cytotoxic and antibacterial activity.

## Material and Methods

### Chemical Agents

RPMI- 1640, Dulbecco’s minimum essential medium (DMEM), fetal calf serum (FCS), trypsin, gentamycin, penicillin, ethylene diaminetetraacetic acid (EDTA), 5-flurouracil, dimethyl sulfoxide, and sulforhodamine-B were purchased from Sigma Chemical Co., USA. All other chemicals were of high purity and obtained locally.

### Plant Material

Whole plant of *C. occidentalis* was collected locally in the month of December and was authenticated at source by the taxonomist of the institute. A voucher specimen has been deposited at the herbarium of the Institute vide IIIM collection No.17687, Acc. No.19361.

### Preparation of Extracts

The authenticated and freshly collected whole plant was chopped and dried under shade. Three extracts of the plant material were made with 95% alcohol, alcohol-water (1:1), and water using repeated solvent extraction procedure. Dried powdered plant material (1 kg) was percolated in 95% alcohol (5 L) at ambient temperature for 16 h. The solvent was decanted and the process was repeated four times. The pooled solvent was evaporated under reduced pressure to yield alcoholic extract (160 g). Similarly, hydro-alcoholic extract was prepared. The dried plant material (200 g) was soaked in alcohol:water (1:1, 1 L) and the extract obtained was 72 g. For aqueous extract the dried powdered plant material (200 g) was heated with distilled water (1.5 L) on steam bath for 2 h, the supernatant was decanted and filtered through celite powder and the process was repeated four times, pooled extract was concentrated on rotavapour and dried in a lyophilizer, and 40 g extract was obtained.

### Cell Lines and Culture

The human cancer cell lines were obtained either from National Center for Cell Science, Pune, India, or National Cancer Institute, Fredrick, USA. The cell lines namely colon (HCT-15, SW-620, COLO 205), ovary (OVCAR-5), prostate (PC-3), and lung (HOP-62) were grown and maintained in RPMI-1640 medium, pH 7.4, whereas DMEM was used for breast (MCF-7) and cervix (SiHa).

### in vitro Cytotoxicity against Human Tumor Lines

The *in vitro* cytotoxicity of extracts was determined using sulforhodamine-B (SRB) as described previously.[16] In brief, the stock solution (20 mg/ml) of the alcoholic, hydro-alcoholic, and aqueous extracts was prepared in dimethylsulfoxide (DMSO), dimethylsulfoxide – water (1:1) and hot water. The stock solutions were further diluted with growth medium (RPMI-1640/ DMEM with 2mM glutamine, pH 7.4, 10% fetal calf serum, 100 *μ*g/ml streptomycin and 100 U/ml penicillin) to obtain desired concentrations. The cells were grown in tissue culture flasks in growth medium at 37°C in an atmosphere of 5% CO _2_ and 95% relative humidity in a CO _2_ incubator. The cells at subconfluent stage were harvested from the flask by treatment with trypsin (0.05% trypsin in PBS containing 0.02% EDTA) and suspended in the growth medium. Cells with more than 97% viability (Trypan blue exclusion) were used for determination of cytotoxicity. An aliquot of 100 *μ*l of cell suspension (10 ^5^ to 2 ×10 ^5^ cells/ml depending upon mass doubling time of cells) was transferred to a well of 96-well tissue culture plate and incubated for 24 h. The test materials (100 *μ*l) were then added to the wells and incubated for another 48 h. The cell growth was stopped by 50 *μ*l of 50% trichloroacetic acid and plates were further incubated at 4°C for an hour. The plates were washed with distilled water and air-dried. Sulforhodamine B (100 *μ*l, 0.4% in 1% acetic acid) was added to each well and plates were incubated at room temperature for 30 min. The unbound SRB was removed by washing with 1% acetic acid and air-dried. Tris-HCL buffer (100 *μ*l, 0.01 M, pH 10.4) was added to all the wells and stirrer. The optical density was recorded on ELISA reader at 540 nm. Suitable blanks and positive controls were also included. Each test was done in triplicate.

The percentage of cell viability was calculated according to the following equation.

$$\mathrm{The}\;\%\;\mathrm{of}\;\mathrm{cell}\;\mathrm{viability}\;=\;\frac{\mathrm{OD}\;\mathrm{of}\;\mathrm{treated}\;\mathrm{cells}}{\mathrm{OD}\;\mathrm{of}\;\mathrm{control}\;\mathrm{cells}}\;\times \;100$$

OD of control cells

### Statistical Analysis

The experiments were repeated three times and the results were expressed as mean ± SD. Statistical analysis was done using unpaired Student’s *t*-test and *P* values < 0.01 were considered significant.

### Antibacterial Assay

The antibacterial assays were performed using the standard protocols.[17]

## Results

### *in vitro* Cytotoxic Effect against Human Cancer Cell Lines

**Figure 1:**
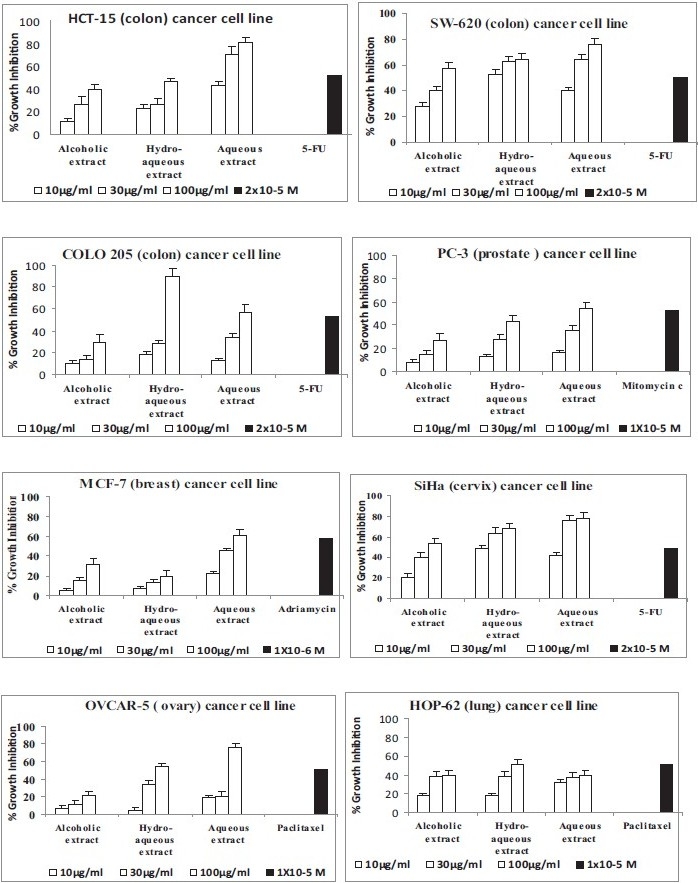
*in vitro* cytotoxicity of C. occidentalis extracts against human cancer cell lines.

*in vitro* cytotoxicity of all the three extracts (alcoholic, hydro-alcoholic and aqueous) of *C. occidentalis* (whole plant) was evaluated at 10, 30, and 100 *μ*g/ml against eight human cancer cell lines from six different tissues origin, namely colon, prostate, breast, cervix, ovary, and lung [Figure 1]. Growth inhibition in a dose-dependent manner was observed in all the cell lines by all there extracts. It was also observed that aqueous extract was most active than hydro-alcoholic and alcoholic extract against all the human cancer cell lines except lung (HOP-62) cancer cell lines where the percent growth inhibition was less than 50%, in comparison to the positive control paclitaxel. When compared with the different positive controls (5-FU, mitomycin C, adriamycin, and paclitaxel) specific for different cell lines the growth inhibition of aqueous extract was found higher than other two extracts. At 100 *μ*g/ml out of eight cancer cell lines aqueous extract showed more than 70% growth inhibition against four cancer cell lines, namely 81% against HCT-15 (colon), 76% against SW-620 (colon), 78% against SiHa (cervix), and 76% against OVCAR-5 (ovary) human cancer cell lines [Figure 1]. Further on lower doses of 30 and 10 *μ*g/ml percent growth inhibition observed by aqueous extract was 71 and 43% against HCT-15 (colon), 64 and 40% against SW-620 (colon), and 76 and 42% against SiHa (cervix) human cancer cell lines. Hydro-alcoholic extract showed highest cytotoxicity against HEP-2, followed by COLO 205 with 96% and 89% growth inhibition at 100 *μ*g/ml, further on lower doses of 30 and 10 *μ*g/ml percent growth inhibition observed was 74 and 39% against HEP-2, 18, and 28 against COLO 205 human cancer cell lines, minimum cytotoxic effect by hydro-alcoholic extract was observed against MCF-7 cancer cell line, and for rest of the cancer cell lines growth inhibition was observed less than 70% at 100 *μ*g/ml. The alcoholic extract showed comparatively less activity against all the eight human cancer cell lines [Figure 1].

### Antibacterial Assay

The *in vitro* antibacterial activity of the three extracts of *C. occidentalis* (whole plant) was found less than that of chloramphenicol, which was used as standard. Out of all the three extracts, hydro-alcoholic extract demonstrated more sensitivity against *Bacillus subtillis* than alcoholic and aqueous extracts. Whereas all the three extracts were found to be inactive against *Escherichia coli, Pseudomonas aeruginosa*, and *Staphylococcus aureus* [Table 1].

**Table 1:** Comparison of *C. occidentalis* extracts on the growth of bacterial species

*Bacterial species*	*Alcoholic extract*	*Hydroalcoholic extract*	*Aqueous extract*	*Chloramphenicol (positive control)*
*Bacillus subtillis*	R	S (9mm)	R	S
*Escherichia coli*	R	R	R	S
*Pseudomonas aeruginosa*	R	R	R	S
*Staphylococcus aureus*	R	R	R	S

S = Sensitive; R = Resistance

## Discussion

The present study observed that the aqueous and hydroalcoholic extracts of *C. occidentalis* inhibits the proliferation of eight human cancer cell lines. The cytotoxicity effect was highest with aqueous extract and least for alcoholic extract. Although the activity demonstrated by the whole plant was low, this may be due to the crude nature of the extracts that can be further enhanced by the purification. The cytotoxicity was concentration-dependent and cell line specific. This clearly indicates the presence of potent bioactive principles in the crude extract that might be useful as antiproliferative and antitumoragents.[18] Although the mechanism of the action have not been elucidated, but there are report that aqueous extracts from leaves of *C. occidentalis* to contain flavonoids and antioxidant polyphenolic compounds.[19] These compounds are known to scavenge the formation of free radicals, have great potential in ameliorating disease processes like cancer and diabetes.[20] The extracts could have exerted its actions by some unknown mechanism ether alone or in combination. Thus the active principle(s) need to be identified.

Similarly, hydro-alcoholic extract of *C. occidentalis* showed potent antibacterial activity against *Bacillus subtillis* strain indicating the presence of chemical constituents responsible for antibacterial activity. The prokaryotic cell structure of a bacterial cell is different than eukaryotic cells. Therefore, both *in vitro* cytotoxicity and antimicrobial bioassays help us to understand the potential activity of a natural compound(s) or a crude extract.[21]

The present study shows that whole plant C. occidentalis possess both *in vitro* cytotoxicity and antibacterial activity. The plant can be further explored for its potential therapeutic uses.

## References

[1] Patwardhan B, Vaidya AD, Mukund Chorghade M (2004). Ayurveda and natural products drug discovery. Curr Sci.

[2] Philip PA (2005). Experience with docetaxel in the treatment of gastric cancer. Semin Oncol.

[3] Austin DJ, Kristinsson KG, Anderson RM (1999). The relationship between the volume of antimicrobial consumption in human communities and the frequency of resistance. Proc Natl Acad Sci U S A.

[4] Kinghorn AD, Su BN, Jang DS, Chang LC, Lee D, Gu JQ (2004). Natural Inhibitors of carcinogenesis. Planta Med.

[5] Kirtikar KR, Basu BD (1987). Indian Medicinal Plants.

[6] Bin-Hafeez B, Ahmad I, Haque R, Raisuddin S (2001). Protective effect of *Cassia occidentalis* L. on cyclophosphamide-induced suppression of humoral immunity in mice. J Ethnopharmacol.

[7] Sharma N, Trikha P, Athar M, Raisuddin S (2000). *In vitro* inhibition of carcinogen-induced mutagenicity by *Cassia occidentalis* and *Emblica officinalis*. Drug Chem Toxicol.

[8] Sadique J, Chandra T, Thenmozhi V, Elango V (1987). Biochemical modes of action of *Cassia occidentalis* and *Cardiospermum halicacabum* in inflammation. J Ethnopharmacol.

[9] Caceres A, Lopez B, Juarez X, del Aguila J, Garcia S (1993). Plants used in Guatemala for the treatment of dermatophytic infections. 2. Evaluation of antifungal activity of seven American plants. J Ethnopharmacol.

[10] Evans CE, Banso A, Samuel OA (2002). Efficacy of some nupe medicinal plants against Salmonella typhi: An *in vitro* study. J Ethnopharmacol.

[11] Tona L, Cimanga RK, Mesia K, Musuamba CT, De Bruyne T, Apers S (2004). *in vitro* antiplasmodial activity of extracts and fractions from seven medicinal plants used in the Democratic Republic of Congo. J Ethnopharmacol.

[12] Badami S, Aneesh R, Sankar S, Sathishkumar MN, Suresh B, Rajan S (2003). Antifertility activity of Derris brevipes variety coricea. J Ethanopharmacol.

[13] Tona L, Mesia K, Ngimbi NP, Chrimwami B, Okond’ahoka, Cimanga K (2001). *In-vivo* antimalarial activity of *Cassia occidentalis, Morinda morindoides* and *Phyllanthus niruri*. Ann Trop Med Parasitol.

[14] Swanston-Flatt SK, Day C, Bailey CJ, Flatt PR (1989). Evaluation of traditional plant treatments for diabetes: Studies in streptozotocin diabetic mice. Acta Diabetol Lat.

[15] Jafri MA, Subhani M, Javed K, Singh S (1999). Hepatoprotective activity of leaves of *Cassia occidentalis* against paracetamol and ethyl alcohol intoxication in rats. J Ethnopharmacol.

[16] Bhahwal AS, Kumar A, Gupta P, Sharma M, Sethi VK, Saxena AK (2007). Cytotoxic and apoptotic activities of novel amino analogues of boswellic acids. Bioorganic and Medicinal Chemistry Letters.

[17] Sahoo S, Kar DM, Mohapatra S, Rout SP, Dash SK (2006). Antibacterial activity of *Hybanthus enneaspermus* against selected UTI pathogens. Indian J Pharma Sci.

[18] Cowan MM (1999). Plant materials as antimicrobial agents. Chem Med Rev.

[19] Meyer BN, Ferrigni NR, Putnam JE, Jacobsen JB, Nicholsand DE, Mclaughlin JL (1982). Brine shrimp; a convenient general bioassay for active plant constituents. Planta Medica.

[20] Sanchez C, Gupta M, Vasquez M, de Noriega, Montenegro G (1993). Bioassay with Artemia to predict antibacterial and pharmacological activity. Rev Med Panama.

[21] Nuhu AA, Aliyu R (2008). Effects of *Cassia occidentalis* aqueous leaf extract on biochemical markers of tissue damage in rats. Trop J Pharma Res.

